# An Interesting Case of a Spontaneous Resolution of Pituitary Adenoma after Apoplexy

**DOI:** 10.5334/jbr-btr.851

**Published:** 2015-09-15

**Authors:** J. Saberifard, T. Yektanezhad, M. Assadi

**Affiliations:** 1 Department of Radiology, The Persian Gulf Nuclear Medicine Research Center, Bushehr University of Medical Sciences, Bushehr, Iran; 2 Department of Neurology, The Persian Gulf Nuclear Medicine Research Center, Bushehr University of Medical Sciences, Bushehr, Iran

A 50-year-old female came to the department in June 2011 with acute onset of severe headache and vomiting associated with visual field defect as bitemporal hemianopia. The symptoms had started acutely and there was no clear history of any such problems in the past. After medical and neurological examination, pituitary adenoma with compression on optic chiasm and raised intracranial pressure was suspected and was confirmed on MRI which showed a large pituitary mass with internal hemorrhage (Fig. A, B). Most of the adenoma was replaced by high signal intensity hematoma on T1 weighted images and there was compression on optic chiasm.

**Figures A–D:**
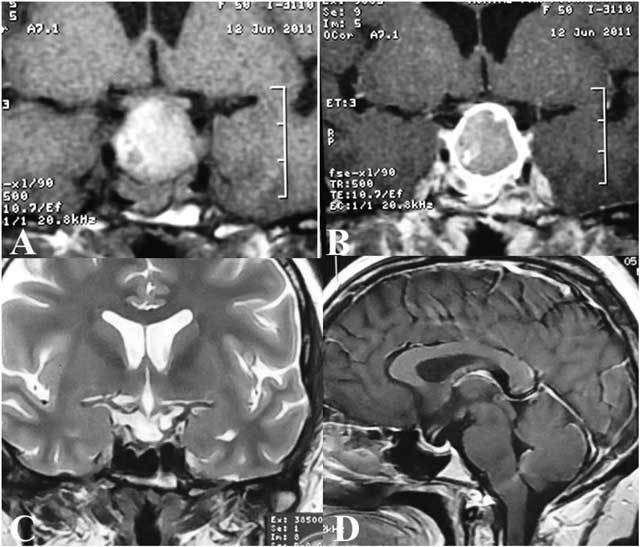


No parasellar invasion of the mass was evident. After contrast injection, only the peripheral portion of adenoma enhanced and most of the central component was non-enhancing which was compatible with necrosis and resultant hemorrhage.

The patient was offered an emergency neurosurgical procedure but she did not accept it and opted for symptomatic treatment by analgesics instead.

She returned two years later for follow-up without any significant symptoms. A follow- up MRI was performed (December 2013) and to our surprise, showed an empty sella without any evidence of pituitary adenoma (Fig. C, D). The pituitary gland showed normal signal intensity and enhancement inside the enlarged sella turcica and no residual adenoma tissue was evident.

## Comment

Pituitary apoplexy is an acute event in pituitary macroadenomas which is defined as massive internal hemorrhage and necrosis. It is a result of infarction, hemorrhage, or hemorrhagic infarction in a pituitary adenoma. Patients usually have preexisting known adenomas but in some cases this may be the presenting picture of previously unrecognized adenomas especially when the adenoma was nonfunctional because most functioning adenomas come to medical attention by endocrinopathies.

The syndrome is characterized by a sudden onset of headache, visual problems, and less commonly altered mental status and sensory or motor dysfunction.

Pituitary hemorrhage occurs more frequently in large tumors which are highly vascular, such as prolactinomas or corticotropinomas. It may also occur in non-functioning adenomas. In cases of non-functioning pituitary tumours (which are ordinarily macroadenomas), a spontaneous disappearance caused by PA is rarely seen.

The prognosis may be poor or good. In cases with better outcome it may result in decrease in the pituitary tumor with or without pituitary hormone deficiency.

The risk of pituitary infarction and hemorrhage is increased in patients receiving anticoagulation medications, oral contraceptive agents, and clomiphene and also after provocative tests for pituitary reserve assessment.

The chance of occurrence of apoplexy is also increased by head trauma, non-pituitary surgery, pregnancy, thrombocytopenia, and increased intracranial pressure.

The exact pathogenesis of PA is not completely understood. Biousse et al identified four groups of so-called precipitating conditions considered as triggering factors for PA.

Classically, pituitary apoplexy is a condition of sudden onset, affecting large neoplasms but Winer and Plant reported sub acute onset of symptoms resembling meningoencephalitis followed by remission in two cases of endocrinologically inactive adenomas.

Clinical pituitary apoplexy is not synonymous with hemorrhage into a pituitary adenoma. Hemorrhage in the pituitary adenoma ranges from small focal hematomas to diffuse hemorrhage throughout the adenoma.

Pituitary is characterized by sudden expansion of the gland due to bland or hemorrhagic infarction. As is evident in our case, most of the pituitary adenoma is non-enhancing on post-contrast images compatible with necrotic component associated with hemorrhage.

Spontaneous remission of endocrinopathy following an apoplectic event is a well known phenomenon in cases with hormonally active pituitary adenomas. Non functioning pituitary adenomas resolving completely following an apoplexy are rare. Spontaneous necrosis of a pituitary adenoma is not rare but represents a very unlikely way of curing a nonfunctioning pituitary adenoma. Most cases cited involve large tumours or have features of Cushing’s syndrome, or acromegaly. The case we presented is a rare outcome of pituitary apoplexy.

## Competing Interests

The authors declare that they have no competing interests.
